# Supplementary figures and images for: Identification of a novel cationic glycolipid in Streptococcus agalactiae that contributes to brain entry and meningitis
Source: PLoS Biol. 2022 Feb 18;20(2):e3001555. doi: 10.1371/journal.pbio.3001555 (PMC8893666; doi:10.1371/journal.pbio.3001555)

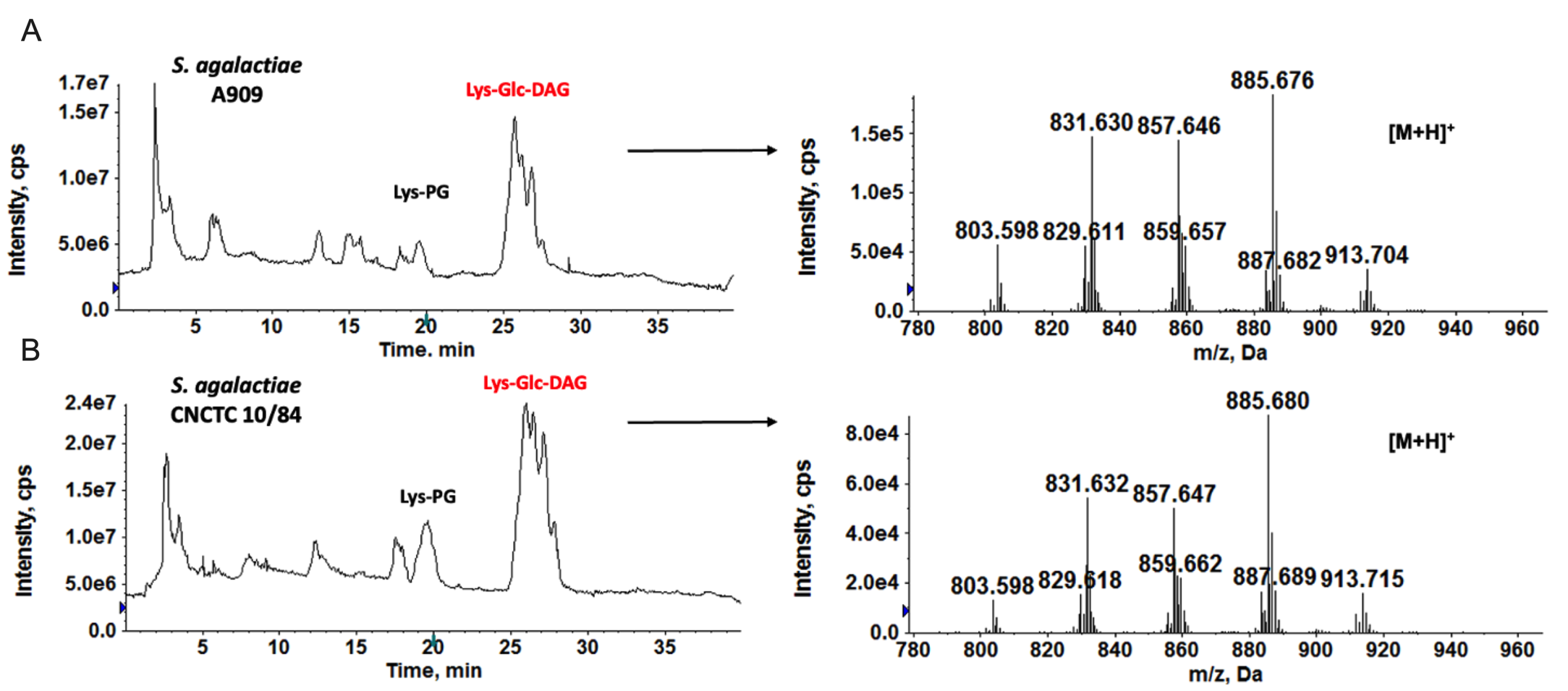

Supplement: S1 Fig — Positive TICs (left panels) showing the presence of Lys-PG and Lys-Glc-DAG in S. agalactiae A909 and S. agalactiae CNCTC 10/84. Mass spectra (right panels) show the [M+H]+ ions of Lys-Glc-DAG. Lys-Glc-DAG, lysyl-glucosyl-diacylglycerol; Lys-PG, lysyl-phosphatidylglycerol; TIC, total ion chromatogram. (TIFF) [file pbio.3001555.s001.tiff]

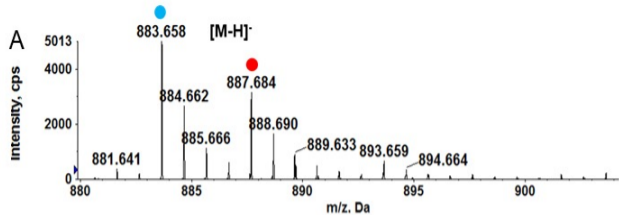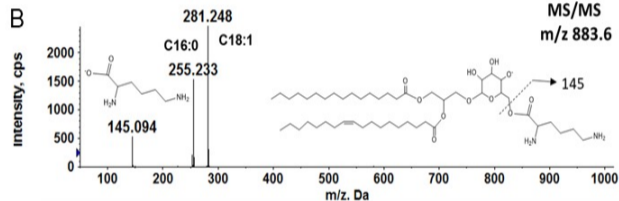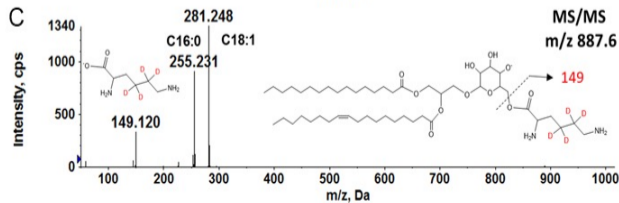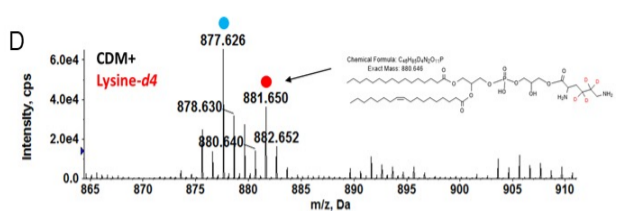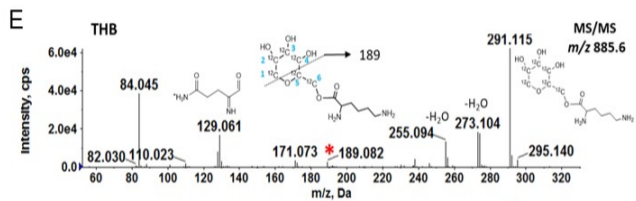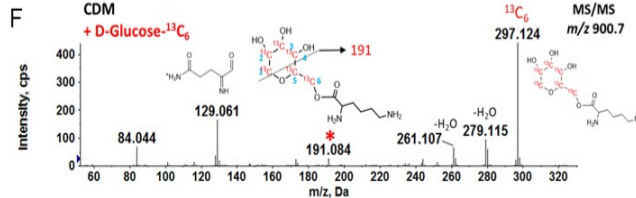

Supplement: S2 Fig — The lipid extracts of S. agalactiae COH1 cultured in DM, DM supplemented with 450 μM L-lysine-d4 (4,4,5,5-D4), or in DM containing 0.5% w/v D-Glucose (U-13C6) were analyzed by LC-ESI/MS in the positive ion mode. (A) Negative ESI/MS of [M-H]− ions of major Lys-Glc-DAG species in S. agalactiae COH1 when cultured in DM supplemented with lysine-d4. The incorporation of lysine-d4 into Lys-Glc-DAG is evidenced by an upward m/z shift of 4 Da of the [M-H]− ion (from m/z 883 to m/z 887). (B) MS/MS of [M-H]− at m/z 883.6 produces a deprotonated lysine residue at m/z 145. (C) MS/MS of [M-H]− at m/z 887.6 produces a deprotonated lysine-d4 residue at m/z 149. (D) [M+H]+ ions of major Lys-PG species in S. agalactiae COH1 cultured in DM supplemented with lysine-d4. The incorporation of lysine-d4 in Lys-PG is evidenced by an upward m/z shift of 4 Da from unlabeled Lys-PG (blue dot) to labeled Lys-PG (red dot). (E) MS/MS of 885.6. A major product ion at m/z 291.1 is derived from glucose-lysine residue. (F) MS/MS of m/z 900.7 (containing 15 13C atoms). The presence of m/z 297.1 (with 6-Da shift) is consistent with glucose in Lys-Glc-DAG is replaced with D-Glucose (U-13C6). The other 9 13C atoms are incorporated into the DAG portion of Lys-Glc-DAG. Furthermore, MS/MS data indicate that lysine is linked to the C6 position of glucose by the fragmentation schemes for forming m/z 189 ion from the unlabeled Lys-Glc-DAG and m/z 191 ion from the 13C-labeled Lys-Glc-DAG. Lys-Glc-DAG, lysyl-glucosyl-diacylglycerol; Lys-PG, lysyl-phosphatidylglycerol; MS/MS, tandem MS. (PDF) [file pbio.3001555.s002.pdf]

**A**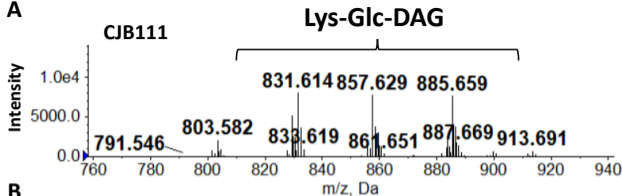**B**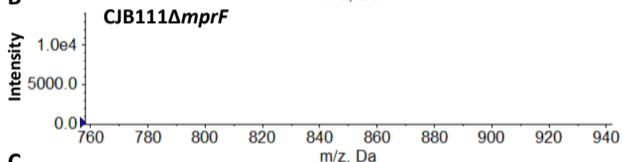**C**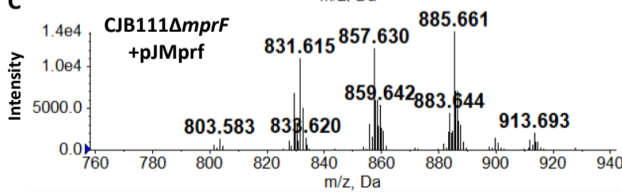

Supplement: S3 Fig — Lys-Glc-DAG is present in the membrane of WT CJB111 (A). Deletion of mprF from CJB111 genome results in loss of Lys-Glc-DAG from the membrane (B). MprF complemented in trans reestablishes Lys-Glc-DAG back into the membrane (C). Lys-Glc-DAG, lysyl-glucosyl-diacylglycerol; WT, wild-type. (PDF) [file pbio.3001555.s003.pdf]

## hCMEC Adherence

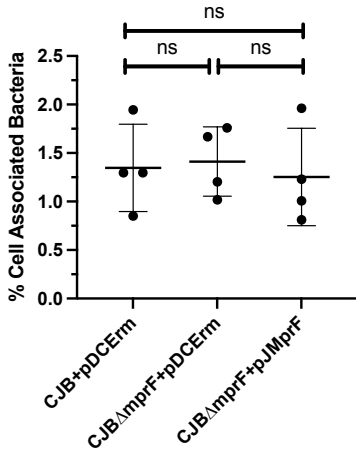

## hCMEC Invasion

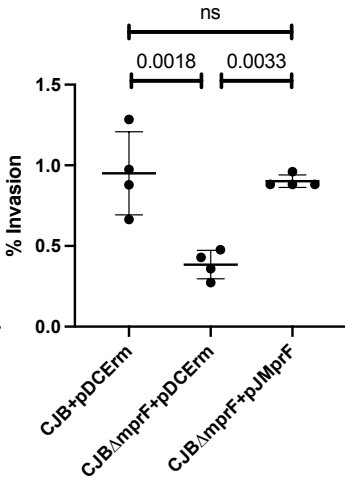

Supplement: S4 Fig — In vitro assays for adherence and invasion of hCMEC cells indicates mprF contributes to invasion but not adherence to brain endothelium. Data indicate the percentage of the initial inoculum that was recovered. Experiments were performed 3 times with each condition in quadruplicate. Data from one representative experiment are shown, mean and standard deviation indicated. One-way ANOVA with Tukey’s multiple comparisons statistical test was used. P-values indicated; ns, not significant. The numerical data underlying the graphs shown in this figure are provided in S1 Data. (PDF) [file pbio.3001555.s004.pdf]
