# Supplementary material for: Identification of a novel cationic glycolipid in Streptococcus agalactiae that contributes to brain entry and meningitis
Source: PLoS Biol. 2022 Feb 18;20(2):e3001555. doi: 10.1371/journal.pbio.3001555 (PMC8893666; doi:10.1371/journal.pbio.3001555)
Supplement: S1 Table — Lys-Glc-DAG, lysyl-glucosyl-diacylglycerol. (DOCX) [file pbio.3001555.s005.docx]

| **Lys-Glc-DAG^1^** | **[M+H]^+^** | |
| --- | --- | --- |
|  | **Observed mass** | **Exact mass** |
| C28:1 | 801.575 | 801.583 |
| C28:0 | 803.595 | 803.599 |
| C30:1 | 829.610 | 829.615 |
| C30:0 | 831.624 | 831.630 |
| C32:2 | 855.623 | 855.630 |
| C32:1 | 857.644 | 857.646 |
| C34:2 | 883.657 | 883.622 |
| C34:1 | 885.672 | 885.677 |
| C36:2 | 911.686 | 911.693 |
| C36:1 | 913.703 | 913.709 |

**S1 Table. Observed and calculated exact masses of the [M+H]^+^ ions of Lys-Glc-DAG molecular species in *S. agalactiae* COH1.**

^1^The numbers before and after colons indicate the total acyl chain carbon atoms and double bonds, respectively.
