# Supplementary material for: Identification of a novel cationic glycolipid in Streptococcus agalactiae that contributes to brain entry and meningitis
Source: PLoS Biol. 2022 Feb 18;20(2):e3001555. doi: 10.1371/journal.pbio.3001555 (PMC8893666; doi:10.1371/journal.pbio.3001555)
Supplement: S2 Table — (DOCX) [file pbio.3001555.s006.docx]

**S2 Table.** **Strains and plasmids used in this study.**

| **Organism** | **Strain** | **Description** | **Ref** |
| --- | --- | --- | --- |
| *S. agalactiae* | ATCC BAA-1176 (COH1) | Wild-type *S. agalactiae* strain, serotype III | (1) |
|  | COH1Δ*mprF* | *mprF* (GBSCOH1_1931) deletion strain | This work |
|  | COH1Δ*mprF(*pABG5) | Empty vector control strain | This work |
|  | COH1Δ*mprF*(pGBSMprf) | Expresses GBS *mprF* from P_prtF_ in pABG5Δ*phoZ* | This work |
|  | COH1(pABG5) | Empty vector control | This work |
|  | CJB111 | Wilt-type *S. agalactiae* strain, serotype V | (2, 3) |
|  | CJB111∆*mprF* | *mprF* (ID870_10050) deletion strain | This work |
|  | CJB111∆*mprF*(pDCErm) | Empty vector control strain | This work |
|  | CJB111∆*mprF*(pJMprF) | Expresses GBS *mprF* in pDCErm | This work |
|  | ATCC BAA-1138 (A909) | Wild-type *S. agalactiae* strain, serotype Ia | (4) |
|  | CNCTC 10/84 | Wild-type *S. agalactiae* strain, serotype V. Obtained from Dr. K Patras, UCSD | (5, 6) |
| *S. mitis* | ATCC 49456 | Wild-type *S. mitis* type strain, also known as NCTC 12261 | (7) |
|  | ATCC 49456(pABG5) | Empty vector control | This work |
|  | ATCC 49456(pGBSMprF) | Expresses GBS *mprF* from P_prtF_ in pABG5Δ*phoZ* | This work |
|  | ATCC 49456(pEfmMprF1) | Expresses *E. faecium mprF1* from P_prtF_ in pABG5Δ*phoZ* | This work |
| *E. coli* | DH5α | Plasmid cloning host; F^–^, φ80*lac*ZΔM15, *rec*A1, *end*A1, *hsd*R17, *pho*A, *sup*E44, λ^–^ *thi*-1, *gyr*A96, *rel*A1 | (8) |
|  | DH5α(pABG5) | Empty vector control | This work |
|  | MC1061 | Plasmid cloning host; F^–^, araD139, ∆(araABC-leu)7696, ∆(lac)X74, galU, galK, hsdR2, (r_K_^-^m_K_^+^), mcrB1, rpsL, (Str^r^) | (9) |
|  | MC1061(pDCErm) | Empty vector control | This work |
|  | DH5α(pGBSMprF) | Expresses COH1 *mprF* (GBSCOH1_1931) from P_prtF_ in pABG5Δ*phoZ* | This work |
|  | DH5α(pEfmMprF1) | Expresses *E. faecium* *mprF1* from P_prtF_ in pABG5Δ*phoZ* | This work |
|  | DH5α(pMBMprFKO) | Allelic exchange plasmid containing ~2 kb sequence flanking GBSCOH1_1931 | This work |
|  | MC1061(pJMprFKO) | Allelic exchange plasmid containing ~2 kb sequence flanking ID870_10050 | This work |
|  | MC1061(pJMprF) | Expresses CJB11 *mprF* from P_tetM/erm_ in pDCErm | This work |
| *E. faecium* | 1,231,410 | Wild type *E. faecium* strain | (10) |
| **Plasmid** | **Description** | | **Ref** |
| pABG5Δ*phoZ* | Constitutive expression vector for streptococci with the P_prtF_ promoter. Confers kanamycin resistance. Referred to as pABG5 throughout the text | | (11) |
| pGBSMprF | pABG5Δ*phoZ* expressing COH1 *mprF* (GBSCOH1_1931) from P_prtF_ | | This work |
| pEfmMprF1 | pABG5Δ*phoZ* expressing *E. faecium* 1,231,410 *mprF1* (EFTG_00601) from P_prtF_ | | This work |
| pMBSacB | Allelic exchange plasmid for *S. agalactiae*. Confers erythromycin resistance and sucrose sensitivity | | (12) |
| pMBMprFKO | Knockout plasmid containing ~2 kb sequence flanking GBSCOH1_1931 | | This work |
| pJMprFKO | Knockout plasmid containing ~2 kb sequence flanking ID870_10050 | | This work |
| pDCErm | Consitituitive expression vector for streptococcus from P_tetM/erm_ | | (13) |
| pJMprF | pDCErm expressing CJB111 *mprF* (ID870_10050) | | This work |
|  |  | |  |

**References**

1. Kuypers JM, Heggen LM, Rubens CE. Molecular analysis of a region of the Group B *Streptococcus* chromosome involved in type III capsule expression. Infection and immunity. 1989;57:3058-65.

2. Faralla C, Metruccio MM, De Chiara M, Mu R, Patras KA, Muzzi A, et al. Analysis of two-component systems in group B Streptococcus shows that RgfAC and the novel FspSR modulate virulence and bacterial fitness. mBio. 2014;5(3):e00870-14.

3. Spencer BL, Chatterjee A, Duerkop BA, Baker CJ, Doran KS. Complete Genome Sequence of Neonatal Clinical Group B Streptococcal Isolate CJB111. Microbiology resource announcements. 2021;10.

4. Lancefield RC, McCarty M, Everly WN. Multiple mouse-protective antibodies directed against Group B Streptococci. Special reference to antibodies effective against protein antigens. The Journal of experimental medicine. 1975;142:165-79.

5. Hooven TA, Randis TM, Daugherty SC, Narechania A, Planet PJ, Tettelin H, et al. Complete Genome Sequence of *Streptococcus agalactiae* CNCTC 10/84, a Hypervirulent Sequence Type 26 Strain. Genome announcements. 2014;2:e01338-14.

6. Wilkinson HW. Nontypable Group B Streptococci isolated from human sources. Journal of clinical microbiology. 1977;6:183-4.

7. Kilian M, Mikkelsen L, Henrichsen J. Taxonomic Study of Viridans Streptococci: Description of *Streptococcus gordonii* sp. nov. and Emended Descriptions of *Streptococcus sanguis* (White and Niven 1946), *Streptococcus oralis* (Bridge and Sneath 1982), and *Streptococcus mitis* (Andrewes and Horder 1906). International Journal of Systematic Bacteriology. 1989;39:471-84.

8. Taylor RG, Walker DC, Mclnnes RR. *E. coli* host strains significantly affect the quality of small scale plasmid DNA preparations used for sequencing. Nucleic acids research. 1993;21:1677-8.

9. Casadaban MJ, Cohen SN. Analysis of gene control signals by DNA fusion and cloning in Escherichia coli. J Mol Biol. 1980;138(2):179-207.

10. Palmer KL, Carniol K, Manson JM, Heiman D, Shea T, Young S, et al. High-quality draft genome sequences of 28 *Enterococcus* sp. isolates. Journal of bacteriology. 2010;192:2469-70.

11. Granok AB, Parsonage D, Ross RP, Caparon MG. The RofA binding site in *Streptococcus pyogenes* is utilized in multiple transcriptional pathways. Journal of bacteriology. 2000;182:1529-40.

12. Hooven TA, Bonakdar M, Chamby AB, Ratner AJ. A Counterselectable Sucrose Sensitivity Marker Permits Efficient and Flexible Mutagenesis in *Streptococcus agalactiae*. Applied and environmental microbiology. 2019;85:1-13.

13. Jeng A, Sakota V, Li Z, Datta V, Beall B, Nizet V. Molecular genetic analysis of a group A Streptococcus operon encoding serum opacity factor and a novel fibronectin-binding protein, SfbX. J Bacteriol. 2003;185(4):1208-17.
