# Supplementary material for: Identification of a novel cationic glycolipid in Streptococcus agalactiae that contributes to brain entry and meningitis
Source: PLoS Biol. 2022 Feb 18;20(2):e3001555. doi: 10.1371/journal.pbio.3001555 (PMC8893666; doi:10.1371/journal.pbio.3001555)
Supplement: S3 Table — (DOCX) [file pbio.3001555.s007.docx]

**S3 Table.** **Primers used in this study.**

| **Primer** | **5’ – 3’ sequence** | | **Use** | |  |
| --- | --- | --- | --- | --- | --- |
| GBS_MprF_F | GAGAGGTCCCTTTCCTTGAAAAAGCTAATTGAAAAAGTC | | Amplify GBSCOH1_1931 for Gibson assembly | |  |
| GBS_MprF_R | ACCAATACCTTTATCTTATTTAACAATCTTAATTTTACTATC | | Amplify GBSCOH1_1931 for Gibson assembly | |  |
| Faec_MprF1_F | GAGAGGTCCCTTTCCTTGTTAAAAAATACCATACAATG | | Amplify EFTG_00601 for Gibson assembly | |  |
| Faec_MprF1_R | ACCAATACCTTTATCTTAATACTTTCTTCGTATCC | | Amplify EFTG_00601 for Gibson assembly | |  |
| MpF_SacII | ACGTCACCGCGGTTGAAAAAGCTAATTGAAAAAGTC | | Amplify CJB111 *mprF* ID870_10050 for ligation | |  |
| MpR_BamHI | ACGTCAGGATCCTTATTTAACAATCTTAATTTTACTATC | | Amplify CJB111 *mprF* ID870_10050 for ligation | |  |
| pABG5-5' | GGAAAGGGACCTCTCTTCCTAAAC | | Linearize pABG5Δ*phoZ* for Gibson assembly | |  |
| pABG5-3' | GATAAAGGTATTGGTAAATAACAAA | | Linearize pABG5Δ*phoZ* for Gibson assembly | |  |
|  | **Expression plasmid sequencing** | |  | |  |
| GBS_S1 | GAATGGAATAATATAGTAGGCT | | For sequencing pGBSMprF/pJMprF, amplifies with pABG5_Fup2/ pF | |  |
| GBS_S2 | GATTGTATCCCTTATTCC | | For sequencing pGBSMprF/pJMprF, amplifies with GBS_S3 | |  |
| GBS_S3 | CGATTCAATAGCTTCAC | | For sequencing pGBSMprF/pJMprF, amplifies with GBS_S2 | |  |
| GBS_S4 | GATAAAAGGCTCTACTGG | | For sequencing pGBSMprF/pJMprF, amplifies with pABG5_FDwn/pR | |  |
| pABG5_FDwn | CCAATAATAATGACTAGAGAAG | | For pABG5 plasmid insert sequencing | |  |
| pABG5_Fup2 | CAAAAGGTTTCGACTTTTCACC | | For pABG5 plasmid insert sequencing | |  |
| EF1_S1 | GAATAACGCTGATCAAAAGT | | For sequencing pEfmMprF1, amplifies with pABG5_Fup2 | |  |
| EF1_S2 | TGCCAAGAGAAATAGTC | | For sequencing pEfmMprF1, amplifies with EF1_S3 | |  |
| EF1_S3 | ACAATCTCTTCGCTTG | | For sequencing pEfmMprF1, amplifies with EF1_S2 | |  |
| EF1_S4 | CCAACTGTTCTTCTCCAA | | For sequencing pEfmMprF1, amplifies with pABG5_FDwn | |  |
| pF | AGCGCTAGGAGGAAAC | | For pDCErm plasmid insert sequencing | |  |
| pR | CCCATGCCATCTCCAATC | | For pDCErm plasmid insert sequencing | |  |
| **GBSCOH1_1931 knockout plasmid construction, sequencing, and integration screening** | | | | |  |
| Mp1F_PstI | ACGTCACTGCAGTTCAATTAGCTTTTTCAACAATTTC | | Amplifies upstream fragment from within GBSCOH1_1931/ID870_10050 leaving 6 codons, with Mp1R_XhoI | |  |
| Mp1R_XhoI | ACGTCACTCGAGGCTGTTTATGGTGCTTTG | | 5’ most primer of upstream fragment, amplifies with Mp1F_PstI | |  |
| Mp2F_XbaI | ACGTCATCTAGAGAAAAGGCTAGATTACGAAC | | 3’ most primer of downstream fragment, amplifies with Mp2R_PstI | |  |
| Mp2R_PstI | | ACGTCACTGCAGGTTAAATAAGCTTTATTTGGCA | | Amplifies downstream fragment leaving 2 codons and stop codon of GBSCOH1_1931/ID870_10050, with Mp2F_XbaI | |
| T7 promoter | TAATACGACTCACTATAGGG | | Amplifies with MpS5F below to sequence plasmid, amplifies with T3 promoter for insert screening and plasmid presence | |  |
| T3 promoter | AATTAACCCTCACTAAAGGG | | Amplifies with MpS3R below, amplifies with T7 promoter for insert screening and plasmid presence | |  |
| Int_F | GCTAATTGAACTGCAGGTTAAATAAG | | Anneals at *mprF* knockout site, amplifies with Out_R for single integration screening | |  |
| Out_R | GCTATTATATTTAGTGGTTTAATTGG | | Anneals outside recombination arms, amplifies with Int_F, for single integration screening | |  |
|  |  | |  | |  |
| **Genomic knockout region sequencing** | | | | |  |
| MpS3F | CATTAGCTAGTCTTATCGGAG | | Anneals outside integration arms, amplifies with MpS3R | |  |
| MpS3R | ACAGCTACTTGGTAGTTCA | | Amplifies with MpS3F | |  |
| MpS4F | GCTACTAAGGCAAGATACG | | Amplifies with MpS4R, knockout screening and plasmid sequencing primer | |  |
| MpS4R | ATGGTCAGCGATGGTG | | Amplifies with MpS4F, knockout screening and plasmid sequencing primer | |  |
| MpS5F | CATAAGCGAAATAACTTGAG | | Amplifies with MpS5R | |  |
| MpS5R | GTATACAACGGCTTGATTGG | | Anneals outside integration arms, amplifies with MpS5F | |  |
